# Assessing the Usability and Feasibility of Digital Assistant Tools for Direct Support Professionals: Participatory Design and Pilot-Testing

**DOI:** 10.2196/51612

**Published:** 2024-04-25

**Authors:** Patrice D Tremoulet, Andrea F Lobo, Christina A Simmons, Ganesh Baliga, Matthew Brady

**Affiliations:** 1 Department of Psychology Rowan University Glassboro, NJ United States; 2 Department of Computer Science Rowan University Glassboro, NJ United States

**Keywords:** technology prototype, data collection, documentation, direct support professionals, intellectual and developmental disabilities, pilot test, mobile phone

## Abstract

**Background:**

The United States is experiencing a direct support professional (DSP) crisis, with demand far exceeding supply. Although generating documentation is a critical responsibility, it is one of the most wearisome aspects of DSPs’ jobs. Technology that enables DSPs to log informal time-stamped notes throughout their shift could help reduce the burden of end-of-shift documentation and increase job satisfaction, which in turn could improve the quality of life of the individuals with intellectual and developmental disabilities (IDDs) whom DSPs support. However, DSPs, with varied ages, levels of education, and comfort using technology, are not likely to adopt tools that detract from caregiving responsibilities or increase workload; therefore, technological tools for them must be relatively simple, extremely intuitive, and provide highly valued capabilities.

**Objective:**

This paper describes the development and pilot-testing of a digital assistant tool (DAT) that enables DSPs to create informal notes throughout their shifts and use these notes to facilitate end-of-shift documentation. The purpose of the pilot study was to assess the usability and feasibility of the DAT.

**Methods:**

The research team applied an established user-centered participatory design process to design, develop, and test the DAT prototypes between May 2020 and April 2023. Pilot-testing entailed having 14 DSPs who support adults with IDDs use the first full implementation of the DAT prototypes during 2 or 3 successive work shifts and fill out demographic and usability questionnaires.

**Results:**

Participants used the DAT prototypes to create notes and help generate end-of-shift reports. The System Usability Scale score of 81.79 indicates that they found the prototypes easy to use. Survey responses imply that using the DAT made it easier for participants to produce required documentation and suggest that they would adopt the DAT if this tool were available for daily use.

**Conclusions:**

Simple technologies such as the DAT prototypes, which enable DSPs to use mobile devices to log time-stamped notes throughout their shift with minimal effort and use the notes to help write reports, have the potential to both reduce the burden associated with producing documentation and enhance the quality (level of detail and accuracy) of this documentation. This could help to increase job satisfaction and reduce turnover in DSPs, both of which would help improve the quality of life of the individuals with IDDs whom they support. The pilot test results indicate that DSPs found the DAT easy to use. Next steps include (1) producing more robust versions of the DAT with additional capabilities, such as storing data locally on mobile devices when Wi-Fi is not available; and (2) eliciting input from agency directors, families, and others who use data about adults with IDDs to help care for them to ensure that data produced by DSPs are relevant and useful.

## Introduction

### Background

In 2019, more than 2 million adults with intellectual and developmental disabilities (IDDs) were living in the United States [1]. Many rely upon direct support professionals (DSPs) for assistance with activities of daily living, such as hygiene, dressing, taking medications properly, eating, accessing and navigating stores, learning vocational skills, participating in therapeutic activities, and socializing [2]. It is widely understood that the quality of life of adults with IDDs is significantly impacted by the quality of the support they receive from DSPs [3-11]. Unfortunately, there has been a shortage in the DSP workforce for more than a decade [7,12]. This shortage and a DSP turnover rate of 44.8% in 2017 led the President’s Committee for People with Intellectual Disabilities to declare a crisis in the direct support workforce [13]. The DSP shortage and high turnover rate, each of which is associated with reduced quality of life for those served by DSPs [3,7], have both significantly increased since the COVID-19 pandemic [14]. Therefore, it is not surprising that the rate of COVID-19 infection among adults with IDDs was disproportionally high and that their quality of life decreased between 2019 and 2020 [15].

DSPs have indicated that generating required documentation is one of the least rewarding and most onerous aspects of their jobs [16]. Several factors make this task challenging, including difficulty recalling details of work performed many hours ago; fatigue; being rushed because employers require them to check out on a time clock at specific times to avoid overtime pay; fear of being accused of copying and pasting content from previous shifts; frequent interruptions by clients or other staff members; and, for some, challenges in writing in a nonnative language [16]. High turnover increases the communication demands on DSPs, including the need to generate detailed documentation to help bring new DSPs up to speed on their clients’ needs. However, overworked DSPs who prioritize clients’ medical and behavioral health needs may struggle to find time to document and share all relevant data during shift changes, unintentionally leaving their clients vulnerable to medical errors and inadequate support [14].

Technology enabling in-the-moment data collection can increase the efficiency of direct support staff and raise the quality of the data they produce, both of which can help improve care for their clients [17-19]. However, most data collection and reporting tools used to capture data about individuals with IDDs are targeted toward providers who work one-on-one with children [16] (eg, paraprofessionals or behavior technicians). These tools are not appropriate for DSPs who support multiple adults with IDDs during their shifts. In addition, the responsibilities of DSPs are much wider in scope than those of paraprofessionals and behavior technicians, who are typically not responsible for tasks such as administering medication and helping prepare meals. DSPs’ responsibilities are closer to those of home health workers who provide older adults or other adults with medical assistance and help with activities of daily living. Many high-income countries are already facing shortages of in-home health workers, while demand for them, as well as for DSPs, is expected to grow in many countries [20-22].

Recognizing that DSPs and the adults with IDDs who depend upon them could both potentially benefit from improved data collection and documentation, the research team used an established user-centered design methodology called Interaction Design and Engineering for Advanced Systems (IDEAS) [23] to design and pilot-test a suite of technology components called a digital assistant tool (DATs) to support data collection and reporting. This methodology relies upon frequent input and feedback from the target users, in this case, DSPs, to ensure that novel technology solutions will be useful, usable, and accepted by the DSPs [23].

### Objectives

The primary purpose of the pilot study described here was to assess the usability of the DAT prototypes. The main objectives of the pilot study were as follows:

1. Identify design inconsistencies and usability problem areas of the DAT.
2. Observe representative users interacting with the DAT prototypes to help assess whether this technology could be effective, efficient, and well received by DSPs.
3. Establish baseline performance and user satisfaction levels in anticipation of more widespread testing of improved versions of the DAT.

## Methods

### Study Design

#### Overview

This project applied the IDEAS methodology, a user-centered participatory design approach that relies upon frequent input and feedback from target users to ensure that novel technology solutions will be useful, usable, and accepted by the users [23]. There are 6 steps in the IDEAS process: needs analysis, requirements generation, design and engineering, interface review, implementation, and evaluation. The first phase of this project included the first 2 steps; the second phase included the third, fourth, and fifth steps; and the third phase, which is the focus of this paper, comprises the last step.

#### Phase 1: Needs Analysis and Requirements Generation

To conceptualize the potential use of technology by DSPs, the research team sought to understand their perspectives on current data collection and documentation techniques and their ideas on how digital technology could be applied to support their work. The results of this exploratory descriptive research, which included focus groups, ethnographic observations, and a survey, are described elsewhere [19]. Using the findings of this formative research, the team developed a list of design principles for our first set of prototypes, shown in Textbox 1.

**Summary of formative research results and the corresponding design principles for the initial digital assistant tool (DAT) prototypes.**

- Direct support professionals (DSPs) need to track and remember large amounts of information about multiple clients.
  The DAT should automatically store notes, it should allow users to associate notes with specific clients, and it should provide access to notes on demand.
- DSPs must continuously monitor clients for safety while tracking behaviors.
  The DAT should enable users to quickly and easily create notes while attending to clients, it should run on mobile and wearable devices, and it should not require a lengthy authentication process.
- DSPs must not allow clients to recognize when DSPs are creating notes about them.
  The DAT should enable DSPs to create notes unobtrusively.
- DSPs are comfortable using smartphones but desire simple, easy-to-use data logging capabilities.
  The DAT should prioritize usability—its user interfaces should be very simple, DAT log-ins should not time out during a shift, and the DAT should provide readable language.
- DSPs do not want employers to be able to access their notes.
  The DAT should feature high levels of security and privacy and allow only DSPs to see their notes.
- DSPs must provide either chronological or categorically organized reports.
  The DAT should time-stamp notes, it should allow users to associate notes with a topic or category, and it should allow users to sort and filter notes.
- DSPs must not copy text from prior days’ reports into the current report.
  The DAT should allow users to copy time-stamped notes into a clipboard or Word document to use to help write reports.

#### Phase 2: Design and Engineering, Interface Review, and Implementation

**Figure 1:**
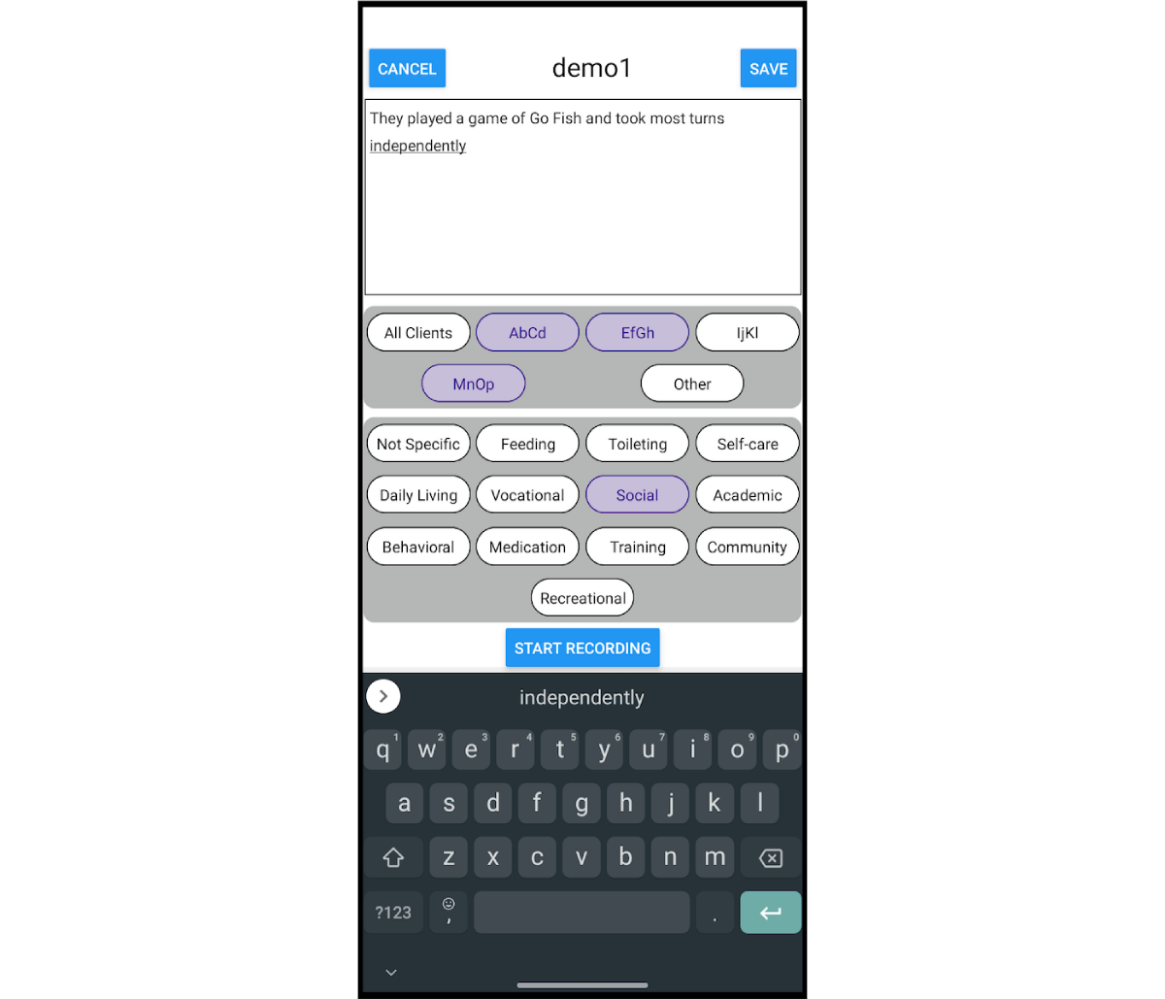
The mobile app, used to create informal notes. This screen shows the touchscreen keyboard used to type new notes.

**Figure 2:**
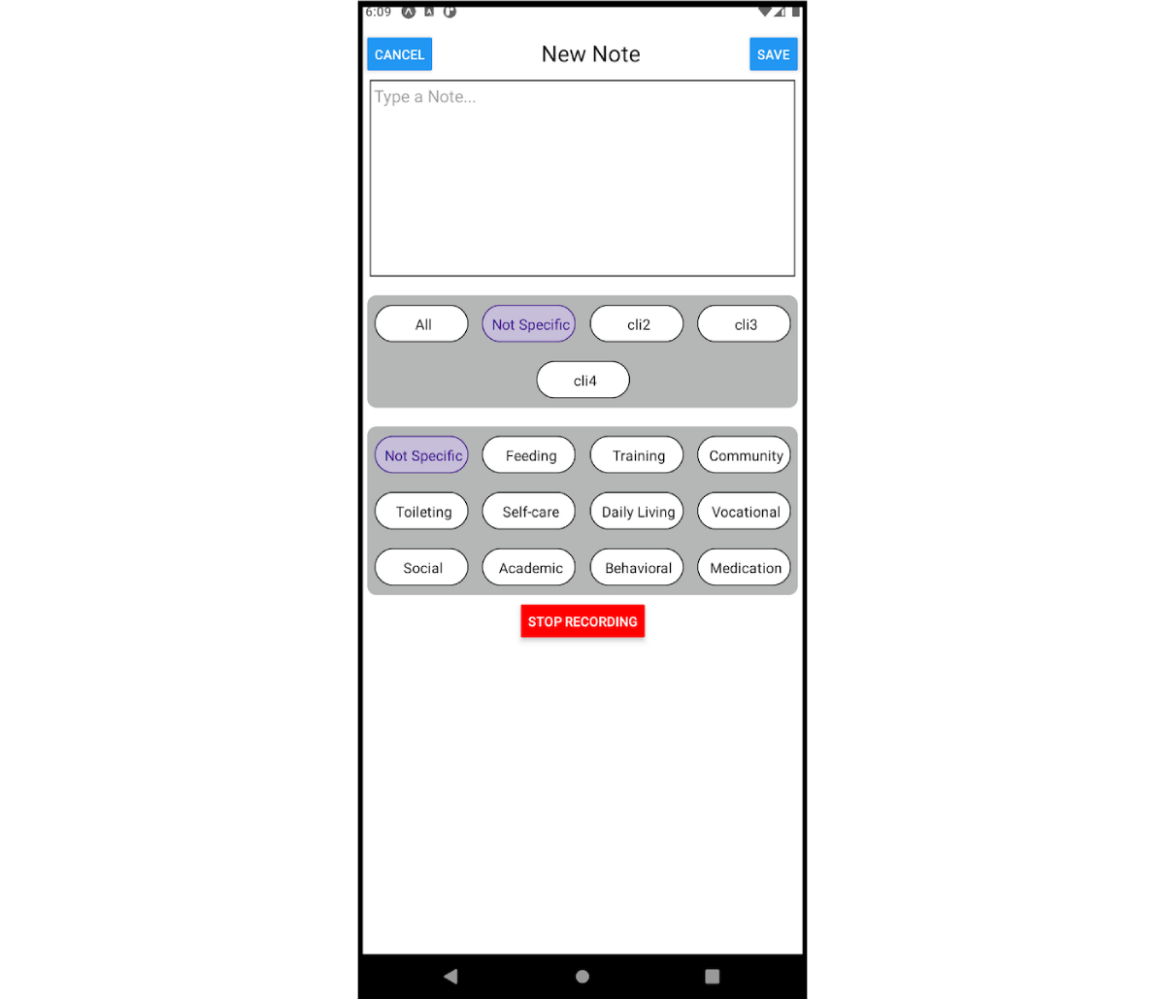
The mobile app, used to create informal notes. This screen shows the screen used for voice-based note creation.

**Figure 3:**
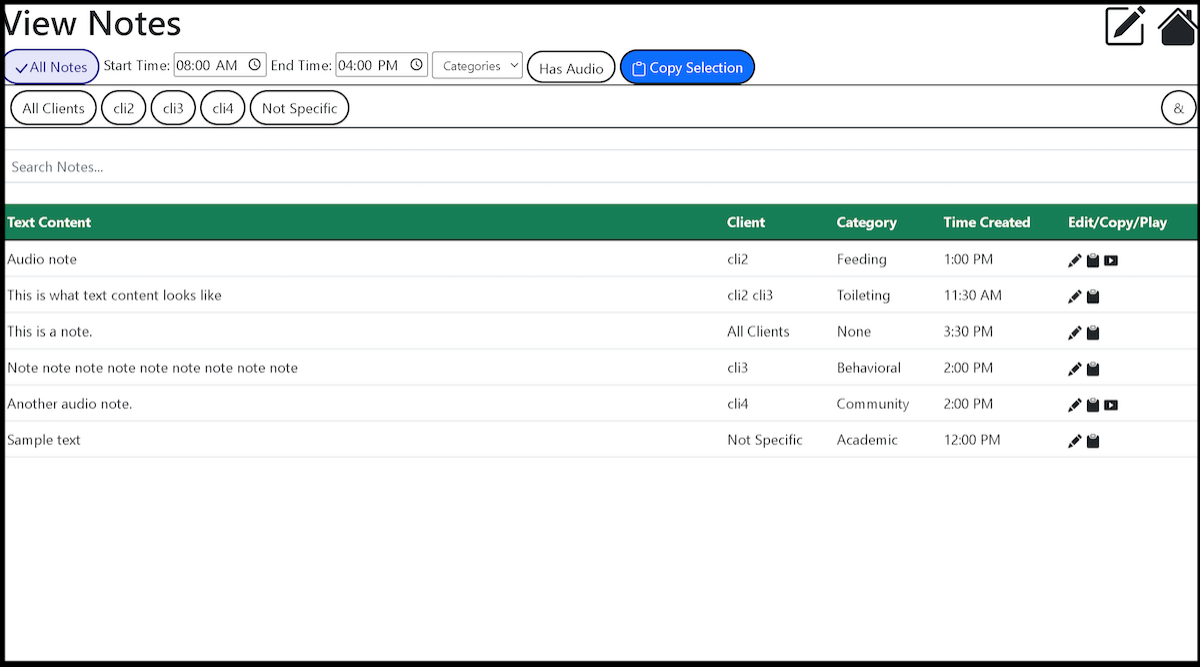
The web application, used to organize notes and copy data into report generation software. cli2: client 2; cli3: client 3; cli4: client 4.

Once the research team had decided on the initial set of capabilities for the DAT, they designed a suite of technology prototypes that (1) enable quick and easy in-the-moment data collection; and (2) allow DSPs to access a private, secure web portal to review, sort, filter, and organize their notes to facilitate end-of-shift documentation. This suite includes 4 components: a mobile app that currently runs on Android smartphones; a private, secure web application that allows DSPs to access, review, and organize notes that they created with the mobile application; a cloud-based center that houses the data; and an administrative website for creating and managing user accounts. As these components were being architected, researchers shared user interface design concepts for the mobile app and the web portal with DSPs to obtain their feedback. The initial wireframes for the mobile app included multiple screens that would allow users not only to create notes but also to review and edit them (Multimedia Appendix 1). After 4 iterations, the team settled upon a very simple single-screen note creation design for the mobile app, also called the Note Creation App; and a spreadsheet-like view of saved notes for the web application, also referred to as the Note Review App (both of which were implemented; refer to Figures 1-3).

#### Phase 3: Evaluation

Once the initial prototypes were implemented, engineering and psychology undergraduate students tested them in laboratory settings; the engineering students focused on performance testing, and the psychology students focused on providing usability feedback. After all known bugs had been fixed, and usability improvements had been implemented, the team began scheduling pilot tests.

### Participants and Recruitment

#### Overview

The pilot study was intended to determine *proof of concept*; therefore, the number of participants was not determined based on traditional power analysis calculations. Guidance on how many participants to include in pilot studies varies, with some recommending between 10 and 30 [24,25] and others suggesting 12 [26,27]. Meanwhile, the System Usability Scale (SUS), the instrument used to measure baseline usability, requires at least 8 to 10 participants to produce reliable results [28]. The research team worked with 3 DSP service provider partners to recruit 16 participants with the goal of having at least 12 (75%) of them test the prototypes during multiple shifts.

The service providers’ senior management members notified their staff via email and during staff meetings that members of the research team would be bringing technology tools designed to be used by DSPs on specific dates. All staff who were working to support adults with IDDs in the designated program for 3 consecutive shifts during the research team visits were eligible to serve as pilot test participants. Due to variability in testing sites, including staff characteristics, the level of support needed by clients served by the staff, and the timing of the staff’s work shifts, demographic data and results are reported separately for each test site.

#### Site 1 (Day Program)

The first pilot-testing site was a day program for adults with IDDS where DSPs and clients were each assigned to 1 of 5 different rooms. Of the 10 DSPs who worked in this setting on the first day of testing, 6 (60%) were selected to serve as pilot testers; 2 (20%) were excluded because they were not scheduled to work 3 shifts in a row in the same setting, and 2 (20%) were willing but unable to participate because there were only 6 smartphones with the DAT mobile app installed available; thus, the first 6 DSPs who volunteered were enrolled. All 6 participants were native English speakers; 5 (83%) were women. Of the 6 participants, 2 (33%) were aged between 18 and 24 years, 1 (17%) was aged between 25 and 34 years, 1 (17%) was aged between 35 and 44 years, and 2 (33%) were aged between 45 and 54 years. All identified as Black or African American. Of the 6 participants, 1 (17%) had a high school degree, while the remaining 5 (83%) had taken some college classes but did not have college degrees. Experience working with adults with IDDs ranged from 1.5 to >20 years, with an average of 8 years, 9.6 months. The amount of time they had held their positions ranged from 4 months to 3 years. Due to last-minute schedule changes, of the 6 participants, 2 (33%) used the DAT prototypes for 2 consecutive shifts, whereas the other 4 (67%) used them for 3 consecutive shifts.

#### Site 2 (Private Home)

The second test site was a private home that housed a single adult with a high level of support needs. Of the 12 different staff members who worked in this home, 4 (33%) were eligible to participate, based on their schedules, and all volunteered to serve as pilot testers. All 4 participants from this site were women and native English speakers; 1 (25%) was aged between 18 and 24 years, and the remaining 3 (75%) were aged between 25 and 34 years. Of the 4 participants, 1 (25%) identified as Black or African American, 1 (25%) as Hispanic, and 2 (50%) as White. Of the 4 participants, 1 (25%) had a high school degree, 2 (50%) had some college but no degree, and 1 (25%) had a college degree. Their experience working with individuals with IDDs ranged from 7 months to 7 years, with an average of 3 years, 11.4 months. The amount of time they had been working in the private home ranged from 3 months to 1.5 years.

#### Site 3 (Provider-Managed Group Homes)

The third test site included 3 group homes, all located in the same county, that housed 3 to 4 adults with IDDs. Each of the 2 staff members in each of the 3 homes volunteered to serve as pilot testers. Of the 6 volunteers, 2 (33%) had work schedule changes that prevented them from using the DAT prototypes after their first shift and were removed from the study; the remaining 4 (67%) volunteers were included in the study. All 4 study participants were women and native English speakers. Of the 4 participants, 3 (75%) identified as Black Hispanic, and 1 as *other*; 1 (25%) had a college degree, and 3 (75%) had high school degrees and no college experience. Of the 4 participants, 1 (25%) was aged between 18 and 24 years, 1 (25%) was aged between 25 and 34 years, and 2 (50%) were aged between 45 and 54 years. The amount of experience they had working with adults with IDDs ranged from 1 to 22 years, with an average of 11 years, 9 months. The amount of time they had held their positions ranged from 1 to 22 years, with an average of 7 years, 4 months. Due to last-minute schedule changes, of the 4 participants, 2 (50%) used the DAT prototypes for 2 consecutive shifts, whereas 2 (50%) used them for 3 consecutive shifts.

### Materials

Each participant was provided with an Android smartphone (Motorola Moto G Power 2021 running Android version 11 RZBS31.Q2-14327-25) that had the DAT mobile app preinstalled. For testing at sites 2 and 3, each smartphone also had shortcuts to all study surveys (demographic survey, post–shift 1 survey, and post–final shift survey) located on its main screen. Shortcuts were not provided on the smartphones during testing at site 1 because the research team was present to provide links in person at that site. All participants used employer-provided laptops to access the DAT web application at the end of their shifts.

### Procedure

On the first day of testing, the principal investigator (PI) obtained informed consent from each participant. She explained that they would be awarded gift cards at the end of the multiday test trial that would be credited with US $75 per shift for their first 2 shifts and US $100 for their third shift. Next, she asked participants to use a computer to complete a short demographic questionnaire, administered through Qualtrics (Qualtrics International Inc; Multimedia Appendix 2).

While participants were filling out the demographic survey, the research team used the administrative website to create user accounts and location-based shifts, which included the initials of the clients whom each participant would be supporting and relevant data categories for the DSPs who worked in this setting (eg, feeding, toileting, behavior, medication, and social skills). These categories were identified by asking staff supervisors to select from a list of possible categories; they were also invited to add items not on the list.

After the participant finished the demographic survey, the researcher showed them the web application, which was populated with sample data previously entered by the research team. The researcher explained to the participant that this application would be used at the end of their shift to support writing the required reports. Subsequently, the researcher provided each participant with an Android smartphone, demonstrated how to use the mobile app, and invited the participants to try creating an audio note as well as a text-based note.

Participants were also invited to review, sort, and filter their sample notes using the web application during the training session. Once participants indicated that they knew how to use both the mobile app and the web application, they were given an opportunity to ask questions and informed that they could request help or address questions to the PI at any point during their shifts. They were then directed to use the smartphone that the research team had provided to log audio and text-based notes about their work while otherwise performing their job as usual throughout their shift.

#### Site 1 Testing

During pilot-testing in the day program, 2 to 3 members of the research team stayed on site during all 3 pilot-testing shifts. One research team member monitored the use of the mobile app through the administrative website, and 2 other members intermittently visited the rooms where the DSPs who were testing the DAT were assigned to work to observe or answer questions. In 1 room, the ratio of DSPs to clients was 1:3 on day 1 and 1:4 on days 2 and 3. In the second room, the ratio of DSPs to clients was 2:5 on day 1 and 2:4 on days 2 and 3. In the third room, the ratio was 2:3 on days 1 and 2 and 3:3 on day 3 (the third DSP in this room was in training and did not have access to the DAT). Finally, the fourth room, which was only included on day 3, had a ratio of 3:8, and only 1 DSP in this room used the DAT.

Throughout all their shifts, the participants used the mobile app to create short notes. They had the option to use voice or a touchscreen keyboard to enter text, and they could also use touchscreen buttons to associate notes with ≥1 clients or to indicate a specific category (eg, feeding, self-care, and medication).

Toward the end of the first day of pilot-testing, when participants were ready to start working on their required reports, a researcher helped them log in to the web application so that they could review and organize the notes that they had created during the shift. If needed, the researcher reviewed functionality, such as sorting, filtering, and copying 1 or multiple notes to a clipboard. The team then observed how the DSPs used the web application to help them create end-of-shift reports. Most of the DSPs (5/6, 83%) decided to paste their notes into either the clipboard or a text file and use 1 of these to draft the text portion of their end-of-shift report, rather than copying note content directly into the application that they use to submit their reports. Once the participants had finished their reports, they were directed to use either the laptop used to create reports or the smartphone to fill out a web-based post–shift 1 survey (Multimedia Appendix 3). Participants were asked to rate 7 items, using a Likert scale ranging from 1 to 5, to indicate their level of agreement. Of the 7 items, 3 (43%) described their experience using the mobile app to log notes, their experience using the web application to review and organize the notes, and their opinions about the quality of the behavioral data and end-of-shift reports that they had provided that day. The items in this survey, which were also included in the final survey, were developed by the authors for this study and were not tested for validity or reliability.

At the end of days 2 and 3, the research team members were available to assist the participants, but all of them were able to access the web application and use it to help them with their reports independently. At the end of day 3, the participants were directed to fill out the final survey (Multimedia Appendix 4). The survey began with 17 items that were rated on a Likert scale ranging from 1 to 5 showing level of agreement. The first 7 items, which were about data collected and reports created, were the same as the first 7 statements rated after the first shift. The last 10 items were those included on the SUS survey, a well-established survey for assessing ease of use and user satisfaction [28,29]. Eleven open-ended questions followed the 17 statements. They asked participants to share their overall impressions of the mobile app and the web application, what they thought about the layout of the mobile app and the web application, what concerns they had about using the DAT, what it was like writing end-of-shift reports while using the DAT, and what additional feedback they wanted to share. These questions were designed to obtain feedback on existing functionality and elicit suggestions for changes or additions.

Once the final survey was submitted, the research team collected the smartphones, and the PI provided gift cards.

#### Site 2 Testing

As the private home housed a single adult, testing at this site was sequential, with only 1 (25%) of the 4 participants using the DAT prototypes on any given shift. In addition, only the PI visited this site when the DSPs who worked there participated in the pilot test. The PI met with each participant in the home approximately 1 hour before their shift started and obtained informed consent, provided the participant with a smartphone running the DAT mobile app, and taught them how to use the DAT mobile app and the DAT web application. Once training was completed, the PI placed shortcuts to the post–shift 1 survey as well as the final survey on the main screen of the smartphone and emailed the participant a private, secure link to the web application site where they could review and organize their notes. Finally, the PI answered any questions that the participant had, made sure the participant had a power cord and knew how to charge the smartphone with the mobile app, provided a phone number and email address to use in case questions or technical difficulties arose during their shifts, and departed. Although the research team members were not on site, they were able to monitor the use of the DAT mobile app and the DAT web application using the DAT administrative portal. All participants used the smartphone to create notes and accessed the web portal at the end of their shifts without support from the research team. Toward the end of each participant’s first shift, the PI sent a reminder email that contained a link to the post–shift 1 survey (which was also accessible via a link on the smartphone). Toward the end of the participants’ third shift, the PI sent an email to confirm a meeting time to deliver gift cards and pick up the smartphones; the email also contained a link to the final survey.

#### Site 3 Testing

Two members of the research team visited each of the 3 group homes during the first day of testing at site 3 to obtain informed consent, hand out the smartphones with the DAT mobile app, and conduct training. The research team members had placed shortcuts to the demographic, post–shift 1, and final surveys on the home screens of the smartphones in advance. After training was complete, the research team members helped participants to create a bookmark to conveniently access the web application on the computers that the participants used to produce their end-of-shift documentation. The PI also emailed links to the surveys and a secure link to access the web application so that the participants could easily access the surveys and their private website for reviewing and organizing their notes from their computers. Next, the researchers gave participants an email address and a phone number that could be used to reach the PI and encouraged them to use these if they had questions or concerns while using the DAT prototypes. The researchers then departed. During the rest of this shift, the research team members monitored the use of the DAT mobile app and the DAT web application via the administrative website. Toward the end of the first shift, the PI emailed participants reminders to fill out the post–shift 1 survey; and toward the end of the third shift, the PI emailed reminders to fill out the final survey. After the third shift had ended for all participants, the PI visited each of the group homes to pick up the smartphones and chargers and distribute gift cards.

### Data Preparation and Analysis

Survey data were extracted from Qualtrics. For each site, descriptive statistics for demographic data were summarized; and the frequencies of the Likert scale responses, except for the 10 SUS items, were computed. The responses to the SUS items from all 14 participants were used to compute a single SUS score, using established computations [29]. The research team members also analyzed open-ended responses from all participants thematically, using inductive open coding [30]. Following the 6-step method formulated by Braun and Clarke [30] as explained by Maguire and Delahunt [31], 2 members of the research team categorized different response types for each question and used these to establish codes; next, these researchers and the PI collaboratively identified high-level themes that ran across multiple questions. Finally, the research assistants independently coded responses to all open-ended questions using the final code set.

In addition, in keeping with the exploratory nature of this work, the research team members analyzed the notes that were collected using the mobile app to understand how DSPs used this technology. In particular, the team reviewed the number of text-based and audio notes that each DSP produced during each of their shifts, the number of words in each type of note, and the number of notes that were started but canceled (not saved) by each DSP during each shift.

### Ethical Considerations

This research complies with the American Psychological Association code of ethics and was approved by the Rowan University Institutional Review Board (PRO-2020-001085). Informed consent was obtained from each participant on the first day of testing. Regarding compensation, at the end of the multiday test trial, participants were awarded gift cards that were credited with US $75 per shift for their first 2 shifts and US $100 for their third shift.

## Results

### Note Logs

Data logs from 34 DSP shifts were imported into Excel (Microsoft Corp) for analysis. Across these shifts, a total of 373 notes were saved. Another 41 notes were started, but they were canceled (not saved). Table 1 shows, for each site, the medians and IQRs of all saved notes per shift, audio notes saved per shift, text-based notes saved per shift, canceled notes per shift, notes created during a DSP’s first pilot-testing shift, notes created during a DSP’s second pilot-testing shift, notes created during a DSP’s third pilot-testing shift, words per audio note, words per text note, and words per note.

**Table 1:** Types of direct support professional notes produced at each site during the pilot-testing.

Computation	Site 1, median (IQR)	Site 2, median (IQR)	Site 3, median (IQR)
Audio notes per shift	2 (1-9)	0 (0)	3 (2-5)
Text-based notes per shift	8 (4-14)	10 (7.5-14)	7 (4-12)
Total notes per shift	10 (5-12)	10 (7.5-14)	10 (2-16)
Canceled notes per shift	0 (0-1.5)	0 (0-3.5)	0 (0-1.5)
Notes created during first shift of pilot-testing	6 (4-12)	7.5 (6-9)	8.5 (5-12)
Notes created during second shift of pilot-testing	10 (5-12)	10 (5.5-14.5)	6 (2-10)
Notes created during third shift of pilot-testing	6 (8.5-12.5)	12.5 (6-16)	9.5 (7-12)
Words per audio note	9 (6-17)	N/A^a^	8.5 (8-14)
Words per text note	12 (6-22.5)	6.5 (3-9)	11 (8-15)
Words per note	10 (6-22)	6.5 (3-9)	10.5 (8-15)

^a^N/A: not applicable.

### Post–Shift 1 Survey Ratings Questions

The post–shift 1 survey began with 7 statements that were rated on a scale ranging from 1 to 5 to show level of agreement (1=strongly disagree, 2=disagree, 3=neutral, 4=agree, and 5=strongly agree). Table 2 shows these statements as well as the frequencies of each of the ratings given to each statement.

**Table 2:** Frequencies of the post–shift 1 survey Likert scale responses for each test site. Response scale ranged from 1=strongly disagree to 5=strongly agree.

Statement	Site 1 (n=6), response; n (%)	Site 2 (n=4), response; n (%)	Site 3 (n=4), response; n (%)
I am confident that today’s data sheets are accurate	4; 1 (17) 5; 5 (83)	5; 4 (100)	3; 2 (50) 4; 1 (25) 5; 1 (25)
I found it easy to record behavior data for all clients today	4; 1 (17) 5; 5 (83)	5; 4 (100)	3; 2 (50) 4; 1 (25) 5; 1 (25)
I believe today’s behavior data will be valuable to others	4; 1 (17) 5; 5 (83)	5; 4 (100)	3; 3 (75) 4; 1 (25)
I found it easy to write session notes today	5; 6 (100)	3; 1 (25) 5; 3 (75)	3; 2 (50) 5; 2 (50)
I am confident today’s session notes contain all necessary information	3; 1 (17) 5; 5 (83)	5; 4 (100)	2; 1 (25) 4; 1 (25) 5; 2 (50)
I am confident today’s session notes contain only relevant information	4; 1 (17) 5; 5 (83)	5; 4 (100)	2; 1 (25) 4; 2 (50) 5; 1 (25)
I believe today’s session notes will be valuable to others (parents, supervisors, behavior analyst)	4; 2 (33) 5; 4 (67)	5; 4 (100)	2; 1 (25) 3; 1 (25) 4; 1 (25) 5; 1 (25)

### Post–Final Shift Survey Ratings and SUS Ratings

All 14 participants who used the DAT during multiple shifts completed the post–final shift survey. Table 3 shows the frequencies of responses to the first 7 statements.

The SUS score for the 14 participants was 81.79, which is quite high for an initial prototype; this score corresponds to *excellent* usability [32]. This score is also in the range where a product is likely to be recommended by users to other potential users [33].

The final survey also contained 11 open-ended questions: 4 (36%) were about the mobile app, 6 (55%) about the web application, and 1 (9%) asked if any initial concerns that participants had about using the DAT prototypes had been alleviated after using them during multiple shifts.

**Table 3:** Frequencies of responses to post–final shift survey non–System Usability Scale Likert scale items.

Statement	Site 1 (n=6), response; n (%)	Site 2 (n=4), response; n (%)	Site 3 (n=4), response; n (%)
I am confident that today’s data sheets are accurate	4; 1 (17) 5; 5 (83)	5; 4 (100)	3; 1 (25) 4; 1 (25) 5; 2 (50)
I found it easy to record behavior data for all clients today	5; 5 (100)	5; 4 (100)	3; 2 (50) 4; 1 (25) 5; 1 (25)
I believe today’s behavior data will be valuable to others	4; 1 (17) 5; 5 (83)	5; 4 (100)	3; 1 (25) 4; 2 (50) 5; 1 (25)
I found it easy to write session notes today	5; 5 (100)	5; 4 (100)	3; 2 (50) 4; 1 (25) 5; 1 (25)
I am confident today’s session notes contain all necessary information	4; 1 (17) 5; 5 (83)	5; 4 (100)	3; 1 (25) 4; 1 (25) 5; 2 (50)
I am confident today’s session notes contain only relevant information	4; 2 (33) 5; 4 (67)	5; 4 (100)	3; 2 (50) 5; 2 (50)
I believe today’s session notes will be valuable to others (parents, supervisors, behavior analyst)	4; 1 (17) 5; 5 (83)	5; 4 (100)	3; 1 (25) 4; 2 (50) 5; 1 (25)

### Thematic Analysis of Open-Ended Question Responses

#### Overview

One-third of the question responses (5/14, 36%) were coded by each of the 2 coders. Intercoder reliability was 92.56%. After coding was complete, the research assistants worked with the PI and identified the following 5 overarching themes in the survey response data.

#### Theme 1: Using the DAT Was a Positive Experience

Participants reported that the mobile app was easy to use and that it was helpful to be able to create notes in real time. Comments after using it for a single shift included “it’s amazing” (Participant 4) and “I would use it on a daily basis” (Participant 12). The participants also provided predominantly positive feedback on the web application, such as “it’s great,” (Participant 2) “simple and easy,” (Participant 7) and “organized and helpful” (Participant 6).

#### Theme 2: Using the DAT Made It Easier to Create End-of-Shift Reports

Survey responses also revealed that participants found that using the DAT facilitated writing end-of-shift reports. Their comments included “[The DAT] made it easy to keep track of everything throughout the day” (Participant 14) and “made [writing reports] a lot easier for me” (Participant 1). A participant wrote, “I had a great experience [writing reports] and I like [the DAT] very much” (Participant 7).

#### Theme 3: The DAT Helped Increase the Accuracy of End-of-Shift Reports

Participants indicated that using the DAT enabled them to create more accurate reports. One wrote, “It help [sic] to maintain accurate notes as the day goes along” (Participant 7); another reported that “It gives you the opportunity to keep the notes accurate” (Participant 9); and a third simply commented, “increases accuracy” (Participant 2).

#### Theme 4: Additional and Improved Features Are Desired

Although participants provided positive responses when asked about their experience using the DAT, they also noted that the tools could provide even greater benefit if they were enhanced. Suggestions included allowing users to access the web application to review and edit notes on their smartphone, improving the transcription accuracy; allowing users to store notes locally when smartphones are not connected to Wi-Fi; offering additional or more specific categories; and allowing users to copy time stamps when copying note text from the web application.

#### Theme 5: More or Better Training Would Help

The survey responses by 3 (21%) of the 14 participants suggested that they would have liked additional training. One of them noted that “it looks easy but when I’m by myself it’s a different story” (Participant 12); the second wrote, “I wish I could get a better understanding” (Participant 11); and the third reported that “it would be easy if I get the hang of it” (Participant 12).

## Discussion

### Principal Findings

All 14 participants were able to use the mobile app without assistance during all shifts, but their ability to use the web application independently at the end of their first shift varied. All participants were able to use both the mobile app and the web application without help from the research team members during their second and third shifts. This result is consistent with an average SUS score of >80, indicating that the DAT prototypes are very easy to use. The hardest part about using the web application for most of the participants was typing in the URLs to access the private, secure notes review app. In future testing, aliases (shorter active links that take users to the same place as the longer URLs) should be set up to help avoid this problem. In addition, research team members should always help participants create a bookmark during training to make it easy to access the web application, a strategy that was not adopted until testing was conducted at site 3. All participants indicated that they found it easier to write reports at the end of their shift after using the DAT.

At site 1, all 6 participants were able to use the DAT prototypes for at least 2 successive shifts. These participants, whose ages were fairly well distributed across the range of 18 to 54 years, agreed or strongly agreed with 6 (86%) of the 7 survey statements after day 1. For the statement “I am confident today’s session notes contain all necessary information,” of the 6 participants, 1 (17%) selected *neither agree nor disagree*, and the remaining 5 (83%) all agreed or strongly agreed (Table 2). When given the same set of questions in the final survey, all 6 participants agreed or strongly agreed with all 7 statements (Table 3). This is consistent with the largely positive responses that these participants provided to the open-ended questions in the final survey. All indicated that they enjoyed using the DAT prototypes and would be interested in using future versions.

The site 2 participants were younger, with ages ranging from 18 to 34 years; therefore, they had less experience working with adults with IDDs than site 1 participants. Their responses to the Likert statements also indicated a high level of agreement; after day 1, all 4 participants strongly agreed with 6 (86%) of the 7 statements; 1 (25%) participant selected *neither agree nor disagree* for the statement “I found it easy to write session notes today,” and the remaining 3 (75%) participants all strongly agreed with this statement. All 4 site 2 participants responded *strongly agree* to all 7 statements in the final survey. These participants also provided responses to the open-ended questions indicating that they enjoyed using the DAT prototypes and would be interested in using future versions. These 4 participants provided more suggestions for additional features than the participants from the other 2 sites.

Site 3 participants fell into 2 different age ranges. Of the 4 participants, 2 (50%) were aged between 18 and 34 years, and 2 (50%) were aged between 45 and 54 years. Compared to site 1 and site 2 participants, site 3 participants provided a wider range of responses to the 2 surveys. As can be seen in Table 2, after the first shift using the DAT prototypes, a participant disagreed with the statements “I am confident today’s session notes contain all necessary information,” “I am confident today’s session notes contain only relevant information,” and “I believe today’s session notes will be valuable to others (parents, supervisors, behavior analyst).” Responses to the other 4 statements ranged from *neutral* to *strongly agree*. Responses to the final survey ranged from *neutral* to *strongly agree* for all 7 statements, indicating more variable sentiments among site 3 participants at the end of the pilot test than among site 1 and site 2 participants.

When we grouped the site 3 participants’ responses based on their ages, we found that those in the age category of 45 to 54 years (2/4, 50%) generally reported a less positive experience using the DAT prototypes than the other participants (2/4, 50%). In fact, a review of the data logs from these 2 participants revealed that they only used the mobile app at the end of their shifts. It seemed that they used the DAT mostly as a transcription service. As the DAT prototypes used a freely available web-based transcription service which was far from perfect, and time stamps are not helpful when all notes are created at the end of shifts, these DSPs would have been better off using a transcription app on the computers they used to create their end-of-shift reports.

While most of the test participants (12/14, 86%) indicated that they valued the ability to collect data about their work in the moment, several of them (6/14, 43%) noted that their ability to benefit from this capability was reduced because the mobile app prototype requires Wi-Fi to save notes. Even with this limitation, the DSPs were very enthusiastic about the DAT prototypes; 1 participant asked whether she could keep using them after the pilot test, and another asked when they would be available for daily use. Future work includes enabling the mobile app to store data locally so that it can be used to collect data while DSPs and clients are out in the community, which is a key part of some DSPs’ client interactions.

The average numbers of notes created per shift and canceled notes per shift was roughly the same for each site. The number of words per note averaged 6.5 at site 2 compared to approximately 10 at sites 1 and 3. To our surprise, audio notes had roughly the same number of words as text-based notes. While the number of words seemed low at first, this is consistent with the intent that note creation be quick, easy, and informal, helping jog memories during report creation. Meanwhile, the average number of notes that DSPs saved during their shifts increased for most DSPs across their shifts. We anticipated that the number of DSPs who created few notes initially would increase in subsequent shifts, and those who created many would create fewer as they learned which types of notes were most helpful. Although 1 DSP’s notes fit this pattern, it seems that the other DSPs created more notes as they became more accustomed to using the mobile app during their shift. In a few cases, participants did not include any content; they just created a note with a client and category selected, indicating that just having a time associated with a client and category would be useful when writing reports. In addition, participants indicated that being able to sort notes by category can directly help with writing reports that have specific requirements, such as detailing all instances of medication administration or all food intake during a shift.

Overall, the results from the pilot test are promising, suggesting that DSPs would be willing to use mobile devices to enable in-the-moment data collection, provided that the collected data facilitate efficient generation of required end-of-shift documentation. Feedback from the test participants suggests that technology such as the DAT could help to increase efficiency, effectiveness, and job satisfaction among DSPs.

### Comparison With Prior Literature

Many sources, some more than a decade old, have warned about, or described, a shortage of DSPs in the United States [7,34-39]. Factors such as heavy workload, onerous documentation requirements, and burnout contribute to a high turnover rate, which exacerbates stress on DSPs [22,40-49]. Some of this literature points out that technology could potentially help to reduce the time DSPs must spend on their other responsibilities, such as documentation, so that they can spend more time on direct support [7,38].

Technology has been successfully applied to increase documentation efficiency and decrease workload in special education and clinic- and home-based therapy programs for children with autism spectrum disorder [17,18,50,51]. By enabling users to quickly and easily record in-the-moment data, these technologies help improve the quality of documentation they generate [52]. However, there is relatively little work that explores how technology could be applied to support direct support workers, such as DSPs and home health aides (HHAs), who provide care to adults [53,54]. Most of the research that addresses having direct support workers leverage technology is qualitative and focuses on workers who support patients without IDDs [55-57]. One 2022 review paper identified only 1 study that created technology expressly intended to support the work of direct support workers [53]. This study, which also entailed creating a smartphone app to use to help create reports, was conducted in Japan nearly 2 decades ago. The authors who described this effort concluded that enabling direct support workers to use smartphones to create reports saves time and reduces costs [58]. Another 2022 review, which surveyed “the technological landscape” of direct support workers, noted that there is a paucity of evidence about how information and communication technologies can be used by these workers [57]. The authors of the review went on to assert that none of the existing technology-based interventions that could be used to facilitate home care were specifically designed to support the workflows of HHAs and concluded that “there is an urgent need for research that centers on the needs and perspectives of HHAs and using human-centered methods to engage HHAs in the design of technologies that truly support their essential caregiving work” [53]. The human-centered research reported here was specifically focused on developing technology for DSPs, but many of the design principles identified in Textbox 1 are also relevant to creating technologies to support the work of direct support workers who care for individuals without IDDs, such as HHAs.

However, inserting technology that is intended to facilitate sharing data among many care providers does not always increase documentation efficiency or decrease workload. Consider the migration from paper to electronic health records (EHRs), which significantly increased clinicians’ workload [59-62]. EHRs have also been associated with a decrease in the amount of direct patient support time [63-65]. In contrast to EHRs, many of which grew out of billing systems [66], the DAT prototypes were developed by following an established user-centered design methodology [23] that ensured that our team considered DSPs’ needs, goals, and constraints and obtained their feedback throughout our effort. As in the case of EHRs, a strong motivation behind creating the DAT prototypes helped to facilitate care for the individuals whose data are being logged electronically [67-70].

Multiple studies have pointed out that clinician burnout, which has been connected to the advent of EHRs, is correlated with decreased patient safety [71-74]. Similarly, DSP burnout and turnover are associated with poor health and quality of life for their clients [3,5]. Hence, technology such as the DAT prototypes, which are designed to reduce the burden of required documentation, improve job satisfaction, and reduce turnover among DSPs, can also help to improve the quality of life of adults with IDDs whom they support.

### Limitations

Several limitations warrant mention. Our pilot test was limited to 14 DSPs working in 1 geographic region. However, different work settings, ranging from a private home with a single resident and group homes with 3 to 4 residents to a day program at a large agency, as well as the range of time that the DSPs who served as pilot testers had worked with adults with IDDs (<1 year to 20 years) increase the generalizability of our results. The pilot test length was also quite short, and we did not measure the amount of time spent generating documentation before the pilot test. These factors prevented us from assessing the impact of the DAT prototypes on documentation efficiency and quality. Nevertheless, the pilot test was long enough to establish feasibility.

Another limitation is that only 10 (71%) of the 14 pilot test volunteers were able to use the DAT prototypes for 3 successive shifts. Scheduling issues, often driven by staffing shortages that caused a staff member to be moved to a different location at the last minute, prevented several participants (4/14, 29%) from using the DAT as planned. In addition, we did not collect data about participants’ familiarity and comfort with technology, although our observations while on site during the first shift provided insights about these factors. The fact that the DSPs were paid to participate is another limitation, although responses to the open-ended questions suggest that at least some of the participants were interested in continuing to use the tools when they would not be paid.

In addition, while the qualitative data from the test users was generally very positive, the study did not use any instruments that assess the likelihood of user adoption, such as the technology acceptance model [75]. Future work on technologies to support DSPs should be informed by the framework developed by Venkatesh et al [76] for understanding user acceptance of IT and might also consider the strategies outlined by Sebastian et al [77] for increasing user acceptance of novel technologies.

### Implications

The results of the pilot test are promising, suggesting that upgraded versions of the DAT prototypes or similar technologies have the potential to reduce the burden of completing end-of-shift reports, while improving the quality of data produced by DSPs. Making it quick and simple to produce time-stamped in-the-moment notes facilitates logging more accurate and more detailed client data. Better data about adults with IDDs would enable family members, health providers, therapists, health providers, and behavior analysts to better support these adults. Long-term benefits of using the DAT could include (1) reducing DSP workload; (2) increasing the time DSPs spend interacting with adults with IDDs; (3) enabling DSPs to provide more consistent and appropriate support; (4) increasing DSP job satisfaction; (5) improving medical and behavioral support for adults with IDDs; and (6) providing a foundation for technology use that increases independence in adults with IDDs, thereby improving their quality of life. Digital documentation could also facilitate timely access to information about adults with IDDs for the diverse stakeholders who help support them.

However, there are risks associated with adopting technology-based data collection tools, including loss of data or an inability to log new data during technology failures. Moreover, to achieve the goal of capturing higher-quality data during work shifts, it will be necessary to allow users to customize the mobile app based on specific clients’ support plans. This will add complexity, which could negatively impact user acceptance because many DSPs do not have a great deal of experience with technology. However, it is possible that supervisors or other employees at agencies that employ DSPs could learn to use an administrative portal to customize the mobile app based on clients’ needs for support.

In any case, it will be important to obtain input from other stakeholders, such as behavioral supervisors, agency directors, and families, in future efforts, particularly as selection of suitable technology is likely the responsibility of the workspace and employer, and communication of data extends beyond DSPs. In the long term, technologies that support data collection will need to be integrated into existing report generation tools. Eventually, these technologies could even leverage artificial intelligence to create first drafts of DSPs’ end-of-shift reports. If this sort of capability is developed, it will be very important to identify strategies for ensuring privacy and security and to consider the ethical implications of using artificial intelligence technologies [78,79].

Finally, while the DAT development effort focused on providing technology that supports DSPs, it is likely that many other types of direct workers could benefit from a similar platform (eg, HHAs, care workers, personal care assistants, certified nursing assistants, and nursing home assistants) [57,80-83]. This is significant because the United States is now facing a severe shortage across the entire direct support workforce [84,85] due in large part to the COVID-19 pandemic. As in the case with DSPs, the shortage in the direct support workforce is harmful to both these workers and those who rely upon them for support [86,87]. A report from the Centers for Disease Control and Prevention revealed that in 2016 a total of 61 million adults (approximately 1 in 4) living in the United States had a disability that impacts major life activities [88]. Many of these adults do, or will eventually, depend upon direct support workers for assistance with activities of daily living.

In summary, future efforts should not only increase the capabilities and robustness of the initial DAT prototypes and consider the needs of family members, medical providers, behavior analysts, and others who would benefit from timely accurate data about adults with IDDs but also explore how these tools could be adapted to meet the needs of other types of direct support workers by eliciting information and feedback from these workers.

### Conclusions

DSPs play a critical role in the care of adults with IDDs. Technology can help mitigate the high turnover rates, poor job satisfaction, and the burden of necessary data collection and documentation that negatively impact DSPs’ ability to care for these adults. The user-centered research effort reviewed here produced proof-of-concept prototypes of tools intended to improve the effectiveness and job satisfaction of DSPs. The results of the pilot test indicate that these tools are likely to provide the intended benefits to DSPs and thus have the potential to help improve the quality of life of clients served by DSPs.

Future research should include testing more robust feature-enhanced versions of the DAT over longer periods in even more diverse settings where DSPs provide support to adults with IDDs. Additional work should also include identifying or developing an instrument to reliably assess report quality and time-motion studies of DSPs before and during longer trials to help quantify how much time DSPs spend generating required documentation. One time-motion study of physicians working on hospital wards found that the physicians believed that they were spending more time on documentation and other administrative tasks than they actually were [89].

In any case, the work reported here, despite its limitations, provides valuable insights into how technology could benefit DSPs and the people they support. Feedback from DSPs indicates that the highest-priority feature enhancement for the DAT prototypes is enabling the mobile app to store data locally to support in-the-moment data collection without Wi-Fi connectivity. Several other enhancements, such as shared task lists, were identified as part of the initial user needs analysis activities performed at the start of this effort [16]. In addition to adding some of these capabilities, future work should identify the needs and constraints of the service providers who employ DSPs to identify barriers to adopting data collection and documentation technologies, such as costs, adaptability in small operations, the need to protect confidentiality, minimizing potential technology damage, and preventing data loss. Such work could help enable future versions of the DAT to supply all caregivers and service providers with the information necessary for better overall service, outcomes, and quality of life for adults with IDDs.

Finally, this line of research needs to be expanded because it could have a profound impact on the health and welfare of several other adults beyond those with IDDs who are supported by direct support workers: older adults, individuals with physical disabilities, and individuals with severe mental illness. Furthermore, the direct support workers themselves could also benefit from technologies such as DATs that enable quick and easy in-the-moment data collection and facilitate end-of-shift reporting.

## Appendix

Multimedia Appendix 1 Initial designs for the digital assistant tool mobile app.

Multimedia Appendix 2 Demographic survey filled out by all pilot test participants.

Multimedia Appendix 3 Post–shift 1 survey filled out after using the digital assistant tool prototypes for a single shift.

Multimedia Appendix 4 Final survey filled out after participants had used the digital assistant tool prototypes for 2 or 3 shifts.

## Abbreviations

DAT: digital assistant tool
DSP: direct support professional
EHR: electronic health record
HHA: home health aide
IDD: intellectual and developmental disability
IDEAS: Interaction Design and Engineering for Advanced Systems
PI: principal investigator
SUS: System Usability Scale
