# Supplementary figures and images for: Assessing the Usability and Feasibility of Digital Assistant Tools for Direct Support Professionals: Participatory Design and Pilot-Testing
Source: JMIR Hum Factors. 2024 Apr 25;11:e51612. doi: 10.2196/51612 (PMC11082739; doi:10.2196/51612)

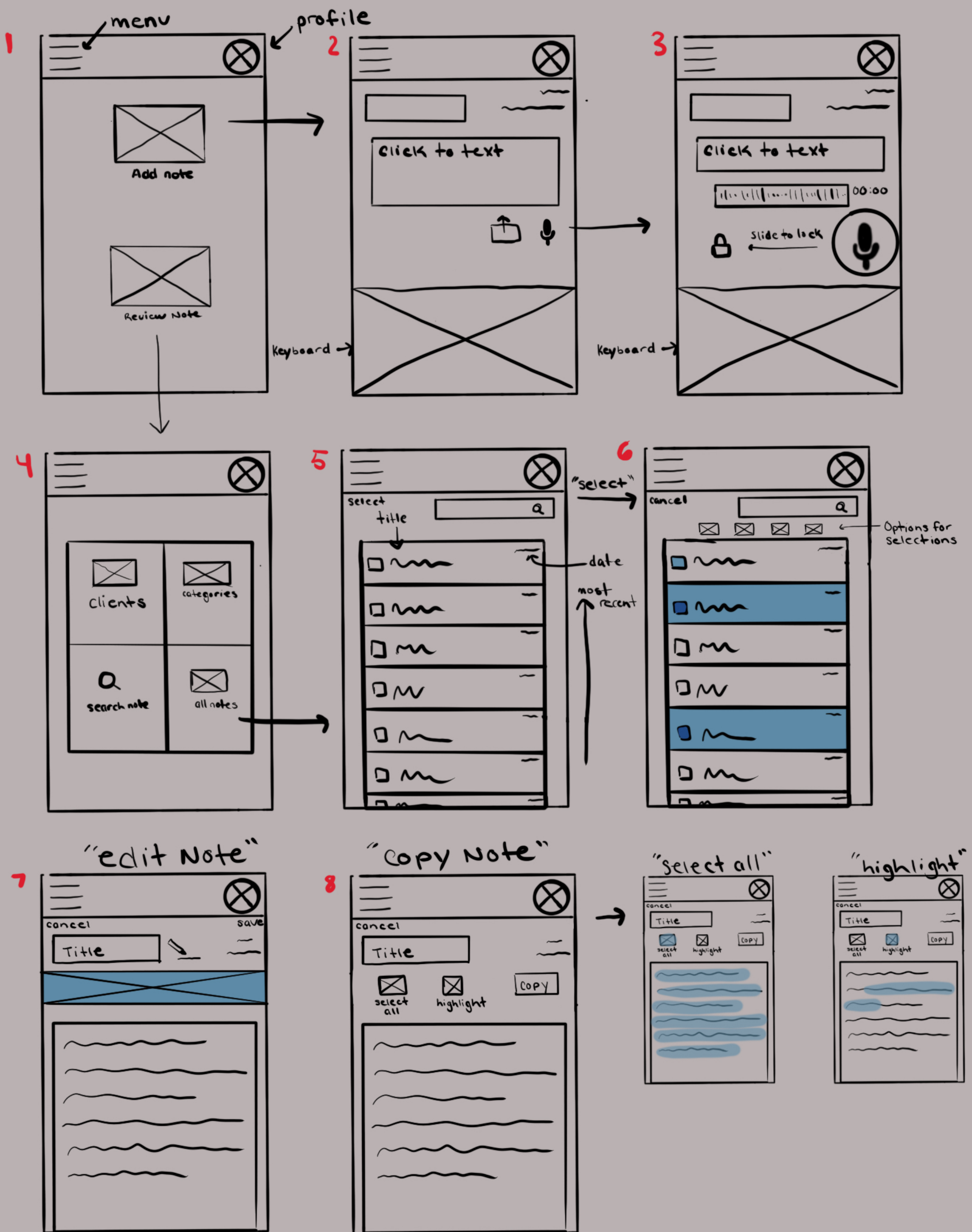

Supplement: Multimedia Appendix 1 [file humanfactors_v11i1e51612_app1.pdf]
