# Supplementary material for: Assessing the Usability and Feasibility of Digital Assistant Tools for Direct Support Professionals: Participatory Design and Pilot-Testing
Source: JMIR Hum Factors. 2024 Apr 25;11:e51612. doi: 10.2196/51612 (PMC11082739; doi:10.2196/51612)
Supplement: Multimedia Appendix 2 [file humanfactors_v11i1e51612_app2.docx]

MM Appendix 2: DAT pilot test Demographic Survey

1. What is your native language? ____________________________________
2. What is your age?

Under 18, 18-24, 25-34, 35-44, 45-54, 55-64, 65-74, 75-84, 85 or older

1. What is your gender?

Male, Female, Other

1. Are you of Hispanic, Latino, or Spanish origin?

Yes, No

1. How would you describe yourself? Please select all that apply.

White, Black or African American, American Indian or Alaska Native, Asian, Native Hawaiian or Pacific Islander, Other

1. How long have you worked with adults with disabilities? _________________________
2. What are your current job responsibilities (check all that apply)

Reduce undesirable behaviors, Teach daily living skills, Teach vocational skills, Teach academic skills, Promote healthy behaviors, Promote social skills, Treat addiction, Train caregivers, Train staff, Assist with feeding, Cleaning/housekeeping chores, Other

1. In which setting(s) do you work (check all that apply)

Residential facility, Day program, Clinic, Home, School, Community

1. How long have you been working in your current job? ___________________________
2. Education What is the highest degree or level of school you have completed?

Less than a high school diploma, High school degree or equivalent (e.g. GED), Some college, no degree, At least one degree post-high school
