# Supplementary material for: Assessing the Usability and Feasibility of Digital Assistant Tools for Direct Support Professionals: Participatory Design and Pilot-Testing
Source: JMIR Hum Factors. 2024 Apr 25;11:e51612. doi: 10.2196/51612 (PMC11082739; doi:10.2196/51612)
Supplement: Multimedia Appendix 3 [file humanfactors_v11i1e51612_app3.docx]

**MM Appendix 3: Post Shift 1 Survey**

(Questionnaire for DSPs at the end of the first shift using the DAT tools)

Please rate level of agreement with statements 1-7 on a 1-5 scale,

where 1 = strongly disagree, 2= disagree, 3 = neither agree nor disagree,

4 = agree, 5 = strongly agree. Then answer questions 8-19.

1. I am confident that today’s data collection sheets are accurate.
2. I found it easy to record behavior data for all clients today.
3. I believe today’s behavior data will be valuable to others.
4. I found it easy to write session notes today.
5. I am confident that today’s session notes contain all necessary information.
6. I am confident that today’s session notes contain only relevant information.
7. I believe today’s session notes will be valuable to others (parents, supervisors, behavior analysts)
8. What do you think of the training video?
9. What do you think of the At-a-glance user guide?
10. What is your overall impression of the note creation app?
11. What do you think about the layout of items in the note creation app?
12. What is your overall impression of the note review website?
13. What do you think about the layout of the note review website?
14. What do you think of the search and filter features of the note review website?
15. What do you think about the wording used for notes copied onto the clipboard?
16. How did you find the experience of writing session notes after using the Digital Assistant tools?
17. Do you anticipate changing how you will use these tools tomorrow?
18. Do you have any concerns about using these tools?
19. Is there any additional feedback you’d like to provide?
