# Supplementary material for: Assessing the Usability and Feasibility of Digital Assistant Tools for Direct Support Professionals: Participatory Design and Pilot-Testing
Source: JMIR Hum Factors. 2024 Apr 25;11:e51612. doi: 10.2196/51612 (PMC11082739; doi:10.2196/51612)
Supplement: Multimedia Appendix 4 [file humanfactors_v11i1e51612_app4.docx]

**MM Appendix 4 Final Survey**

(Questionnaire for DSPs at the end of the last (third) shift of the pilot test)

Please rate level of agreement with the statements numbered 1-17 on a 1-5 scale,

where 1 = strongly disagree, 2= disagree, 3 = neither agree nor disagree,

4 = agree, 5 = strongly agree. Then respond to questions 18-25.

1. I am confident that today’s data collection sheets are accurate.
2. I found it easy to record behavior data for all clients today.
3. I believe today’s behavior data will be valuable to others.
4. I found it easy to write session notes today.
5. I am confident that today’s session notes contain all necessary information.
6. I am confident that today’s session notes contain only relevant information.
7. I believe today’s session notes will be valuable to others (parents, supervisors, behavior analysts).
8. I think that I would like to use this system frequently.
9. I found the system unnecessarily complex.
10. I thought the system was easy to use.
11. I think that I would need the support of a technical person to be able to use this system.
12. I found the various functions in this system were well integrated.
13. I thought there was too much inconsistency in this system.
14. I would imagine that most people would learn to use this system very quickly.
15. I found the system very cumbersome to use.
16. I felt very confident using the system.
17. I needed to learn a lot of things before I could get going with this system.
18. What did you like best about the note creation app?
19. What did you like least about the note creation app?
20. What did you like best about the note review website?
21. What did you like least about the note review website?
22. What do you think of the search and filter features of the note review website?
23. What changes would you recommend to either the app or website?
24. What concerns do you have about using these tools?
25. Were any concerns you had at the start of day 1 alleviated by the end of day 3?
